# The Use of Bayesian Networks to Assess the Quality of Evidence from Research Synthesis: 1.

**DOI:** 10.1371/journal.pone.0114497

**Published:** 2015-04-02

**Authors:** Gavin B. Stewart, Julian P. T. Higgins, Holger Schünemann, Nick Meader

**Affiliations:** 1 Centre for Reviews and Dissemination, University of York, York, United Kingdom; 2 School of Social and Community Medicine, University of Bristol, Bristol, United Kingdom; 3 Department of Clinical Epidemiology & Biostatistics, McMaster University Health Sciences Centre, Hamilton, ON, Canada; University of Illinois-Chicago, UNITED STATES

## Abstract

**Background:**

The grades of recommendation, assessment, development and evaluation (GRADE) approach is widely implemented in systematic reviews, health technology assessment and guideline development organisations throughout the world. A key advantage to this approach is that it aids transparency regarding judgments on the quality of evidence. However, the intricacies of making judgments about research methodology and evidence make the GRADE system complex and challenging to apply without training.

**Methods:**

We have developed a semi-automated quality assessment tool (SAQAT) l based on GRADE. This is informed by responses by reviewers to checklist questions regarding characteristics that may lead to unreliability. These responses are then entered into the Bayesian network to ascertain the probabilities of risk of bias, inconsistency, indirectness, imprecision and publication bias conditional on review characteristics. The model then combines these probabilities to provide a probability for each of the GRADE overall quality categories. We tested the model using a range of plausible scenarios that guideline developers or review authors could encounter.

**Results:**

Overall, the model reproduced GRADE judgements for a range of scenarios. Potential advantages over standard assessment are use of explicit and consistent weightings for different review characteristics, forcing consideration of important but sometimes neglected characteristics and principled downgrading where small but important probabilities of downgrading are accrued across domains.

**Conclusions:**

Bayesian networks have considerable potential for use as tools to assess the validity of research evidence. The key strength of such networks lies in the provision of a statistically coherent method for combining probabilities across a complex framework based on both belief and evidence. In addition to providing tools for less experienced users to implement reliability assessment, the potential for sensitivity analyses and automation may be beneficial for application and the methodological development of reliability tools.

## Introduction

The fundamental objective of evidence-based health care is to enable clinicians or policy makers to make informed decisions regarding the development or delivery of effective health interventions. The reliability of the evidence underpinning decisions is important particularly where high and low quality evidence result in different conclusions regarding effectiveness. Researchers have developed a range of tools for considering the validity of conclusions from research synthesis. Easily applicable tools [e.g. 1] focus on the process and robustness of research synthesis but their practical utility is limited as they do not provide direct information on the quality of evidence underpinning decisions. This requires evaluation of biases across studies, as well as biases detected during the process of evidence synthesis.

The Grades of Recommendation, Assessment, Development, and Evaluation (GRADE) approach [2–16] is the most widespread method for rating the quality of evidence in healthcare. GRADE has been adopted by organisations such as the World Health Organization, Cochrane Collaboration, Agency for Healthcare Research and Quality, and National Institute for Health and Clinical Excellence.

The GRADE approach is described in online support for software [http://ims.cochrane.org/revman/gradepro] and in a *BMJ* series of papers in 2008, with updated guidance available in a *Journal of Clinical Epidemiology* series beginning in 2011 [2–16]. According to the GRADE approach, evidence based on randomised controlled trials is considered high quality (reliable), but can be downgraded across five domains: risk of bias, inconsistency, indirectness, imprecision, publication bias. Non-randomised studies begin at low quality evidence but their rating can be upgraded (provided no other limitations have been identified in the five domains) for three primary reasons: large magnitude of effect, evidence of a dose-response effect, all plausible confounding taken into account. This then leads to a rating on the quality of evidence for each outcome of high, moderate, low or very low where high indicates that any future research is likely to be confirmatory.

A key advantage of GRADE is that it leads to transparent judgments on the quality of evidence. Proposing key specific factors that may lead to downgrading the quality of evidence (and the need to state reasons for each downgrade) provides a clear rationale for such judgements. Another desirable feature of the approach is the ability to make complex, nuanced judgements using a common framework that is not based solely on review process or the type of evidence included in the synthesis [17].

GRADE is not without detractors [18]. Specific criticisms include failure to adequately account for dependencies across domains, and lack of nuanced judgements when combining multiple sources of bias to produce an overall judgement on the strength of evidence. These criticisms partly reflect limitations in current knowledge. Many aspects of GRADE are consensus based due to limitations in the current evidence base and the approach continues to be modified and updated in the light of new evidence from methods research.

The widespread acceptance of evidence-based health care means that systematic reviews are widely viewed as gold standard evidence. Uncertainty associated with reviews of poor quality studies, with poorly framed questions or employing poor methodology is often underestimated. Rating the strength of a body of evidence is a key factor in translating to clinicians and decision-makers the confidence with which they should interpret these results [19]. Although we acknowledge such judgements will always require a certain level of subjectivity, reviewers should be expected to meet a reasonable level of consensus particularly in the context of evidence based decision making which is founded on the principles of transparency and scientific rigour. In this paper we propose a quality assessment tool using Bayesian networks to formalise the structure provided by the GRADE framework, and explicitly weighting the different items that combine to reduce overall strength of evidence. This will facilitate transparent and consistent application of GRADE and communicate uncertainty in the assessment which is currently lacking. Such networks still require value judgements in parameterisation and will likely provide less nuanced judgements than those made by experienced methodologists, but may increase the repeatability of assessment making widespread application by non-specialists feasible. They could also lead to the automation or semi-automation of quality assessments.

Bayesian networks evolved in the early 1990s based on the seminal works of researchers such as Pearl, Lauritzen, Dawid and Spiegelhalter. Bayesian networks (sometimes referred to as causal diagrams, directed acyclic graphs or DAGs) are statistical models of a domain, comprising a network of nodes connected by directed links, with a probability function attached to each node [20]. They are often used to model uncertainty, and therefore provide a tool for decision-making under uncertainty [20]. This uncertainty can arise owing to an imperfect understanding of the domain, incomplete knowledge of the state of the domain, randomness in the mechanisms governing the behaviour of the domain, or any combination of these [21,22]. Previously proposed tools have assessed quality in control processes [23,24]. Here we suggest a tool based on Bayesian networks can bring the advantages of transparency, reliability and statistical coherence to the assessment of reliability of evidence from research syntheses.

## Development of the Bayesian Network

We reviewed the updated guidance in the *Journal of Clinical Epidemiology* [2, 16] on downgrading evidence from meta-analysis based on randomised controlled trials, and deconstructed the information underlying the five domains. Two authors [GS, NM] developed a checklist of questions underpinning the GRADE approach based on the JCE series of articles with differences in interpretation resolved through discussion (supplementary material checklist and [17] for further details).

**Fig 1:**
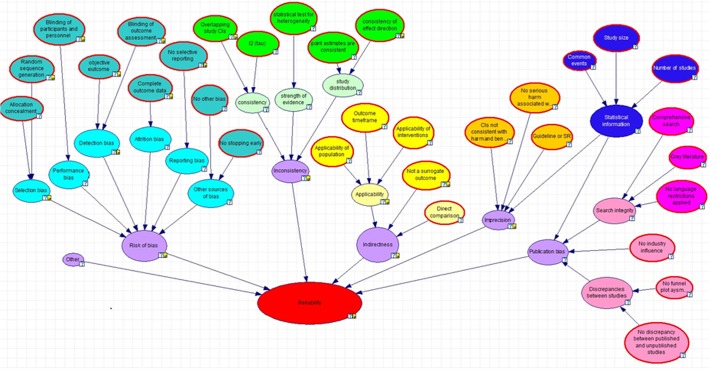
A logic map depicting the GRADE framework for assessing strength of evidence. (This formulation considers risk of bias only for randomised control trial study designs).

The same two authors built a casual diagram explicitly mapping the relationships between questions and domains (Fig. 1). For example, we ask a question about allocation concealment and a question about random sequence generation to ascertain the probability of selection bias (S1 Table). The structure of the causal diagram was checked for consistency with empirical evidence and the GRADE approach by two other authors [JPTH, HS] with any differences in interpretation resolved through discussion. Two authors [GS, NM] then attributed probabilities to each domain, conditional on the answers to the questions informing it. For example, if there was no allocation concealment and inadequate random sequence generation then the probability of selection bias being realised (high) was judged to be 1.0 (S1 Table). These probabilities were elicited through discussions between the two authors and reading the GRADE literature. Any differences in proposed probabilities were resolved through discussion or consultation with a third author [JPTH]. We recognise that all the probabilities throughout the paper are (reasonably arbitrary) proposals reflecting either logic or empirical evidence and expert consensus regarding threats to validity of research synthesis. They can easily be amended to reflect alternative judgements or evidence [25]. The rationale for the assignment of probabilities for each domain is outlined below.

Reviewers wanting to use the tool to obtain a judgement on the strength of evidence answer the checklist questions (for the full checklist of question please see supplementary materials, or for further details see [17]). These responses to the checklist questions are entered into the Bayesian network which then estimates the probability of there being no limitation, serious limitations, very serious limitations (i.e. no downgrade, downgrade one level, downgrade two levels for the GRADE assessment). The final judgement of strength of evidence is then estimated by the Bayesian network based on the estimates of bias/limitations in each of the five domains (i.e. risk of bias, imprecision, indirectness, inconsistency, publication bias).

### Risk of bias

For the risk of bias associated with randomised trials we referred to guidance from the Cochrane Collaboration [26] in addition to the GRADE literature. Selection bias was based on allocation concealment and random sequence generation, which were given equal weight (S1 Table). Performance bias was determined solely by blinding (S2 Table). Detection bias was based on blinding and whether the outcome was objective, being most likely when a subjective outcome was measured unblinded (S3 Table). The severity of attrition bias depends on the event rate in relation to losses to follow up with low ratios being problematic. We used an arbitrary value for loss to follow up of 20% as a surrogate measure (S4 Table). Reporting bias was related to outcome reporting (S5 Table). An additional “catch all” item was used to capture other biases (e.g. baseline imbalance) and early stopping (S6 Table). This framework represents a highly simplified variant of the more nuanced guidance provided by the Cochrane Collaboration [26]. We used a simple decision rule to derive the conditional probabilities underlying risk of bias. Selection bias results in serious limitations, or very serious limitations if combined with a problem from any alternative source; two problems from other sources (e.g. detection bias, attrition bias) result in serious limitations, whilst three problems result in very serious limitations (S7 Table). The option of answering questions as unclear results in no downgrading (the probabilities are equivalent to the “low” state) but their inclusion allows users to distinguish judgements regarding absence of bias from judgements regarding absence of evidence of bias. Where unclear judgements are intermediate between alternative options, an alternative parametisation may be appropriate if a more conservative approach to assessing poorly reported reviews is desired.

### Inconsistency

Inconsistency is the extent to which individual study effects differ. The determinants of inconsistency are substantial variation in effects across studies (in both magnitude and direction), and sufficient information within studies to ascertain that differences are not due to chance.

Consistency is assessed using a statistical measure of the proportion of detectable between study variation that is not due to chance (I^2^) whilst the degree of overlap of study confidence intervals provides a heuristic interpretation. In our schema the latter carries more weight than the former with probabilities for “some overlap” intermediate between the values assigned to “substantial overlap” and “no overlap” (S8 Table). The extent of evidence for heterogeneity can be measured in terms of the statistical significance of the heterogeneity test (S9 Table). An unclear judgement here is intermediate, in contrast to the risk of bias domain. ‘Study distribution’ considers whether there is meaningful variation in study effects and variation in effect direction. Inconsistency is more likely to be substantial when there is variation in both, whilst variation in direction is unimportant if there is not judged to be meaningful variation in effects (S10 Table). Overall inconsistency is serious to very serious if study distributions are wide, with consistency and amount of information carrying less weight (S11 Table).

### Indirectness

Assessing the applicability or indirectness of evidence involves many difficult value judgements regarding the match between the available evidence and the decision setting (which may itself vary) or research question. The GRADE literature suggests assessing how directly the body of evidence relates to the question asked, in terms of population, intervention, comparator, and outcome scale or outcome time frame. In that framework the use of surrogate outcomes is a major cause of indirectness (S12 Table). Another determinant of indirectness is the nature of the comparison, with direct comparison of active interventions generally considered more robust than indirect comparisons via a common comparator (S13 Table). Overall indirectness is likely very serious when surrogate outcomes and other indirectness, such as lack of population directness are combined, with either alone likely to be serious (S13 Table).

### Imprecision

Imprecision refers to uncertainty in the magnitude of the point estimate, typically expressed using 95% confidence intervals. Where the confidence interval is consistent with both benefit and harm, imprecision is high. In the context of guideline development, if alternative management decisions would be made based on utilising the effects compatible with those reflected in the CI, then the consequences of imprecision are more serious, warranting further downgrading. Whether confidence intervals are consistent with benefit and harms depends partly on the effectiveness of the intervention and partly on the amount of statistical information, which in turn depends on choice of meta-analytical model, study size, number of studies, event rates and heterogeneity. Given the complexity of the relationships between these components, we chose to consider statistical information as a function of the size of studies, number of studies and event rate (for dichotomous outcomes) (S14 Table). Overall imprecision was likely to be very high where statistical information was low and the summary effect was consistent with benefit and harms (as defined using a minimally important difference) (S15 Table).

### Publication bias

Publication bias is determined by a combination of discrepancy between published and unpublished studies, amount of statistical information, industry influence and search integrity with the former carrying greatest weight (S16 Table). Search integrity is determined by the comprehensiveness of the search, inclusion of grey literature and the application of any language restrictions (Fig. 1). The discrepancy between studies is informed by funnel plot asymmetry in addition to subjective assessment (Fig. 1). In contrast to the other domains where bias is classified as no bias, serious bias or very serious bias, publication bias is either not detected or strongly suspected reflecting the inherent uncertainty of identifying this type of bias (S15 Table).

### Combining information across domains

GRADE generally uses a considered judgement across domains to determine overall quality of evidence, with serious limitations in any domain resulting in a downgrade of one category (e.g. high to moderate). We used conditional probabilities of zero or one to implement the GRADE framework determining overall quality of evidence [http://ims.cochrane.org/revman/gradepro]. Two serious limitations or a very serious limitation within one domain result in a downgrade of two categories (e.g. high to low), whilst three serious limitations or one serious and one very serious limitations always results in a very low quality rating of evidence.

### Application of the tool

We considered five scenarios to illustrate the performance of the tool when rating the quality of systematic review evidence.

Scenario one: We considered a hypothetical “high quality” review of fifteen large randomised controlled trials where the blinding of participants was unclear and there was some heterogeneity but no other problems were encountered.

Scenario two: This is identical to scenario one, but the review evidence comes from three small randomised controlled trials.

Scenario three: This is identical to scenario one, but additional problems are identified with use of a surrogate outcome and inadequate randomisation methods

Scenario four: We considered a meta-analysis of seven moderate sized studies where reporting risk of bias was unclear, heterogeneity was high, effect direction was inconsistent and all aspects of the search for evidence were poor.

Scenario five: We considered a meta-analysis of five small studies, with unclear reporting of risk of bias, substantial variation in effect magnitude and direction but substantial overlap of individual study confidence intervals, high heterogeneity, surrogate outcome and industry funding.

We also randomly selected four Cochrane systematic reviews [27–30] with GRADE assessments and compared overall assessments of quality presented in summary of findings tables with the results from the Bayesian network to provide an initial proof of principle.

The five scenarios illustrate strong congruence between the judgements derived from the tool and a GRADE assessment based on guidance from the GRADE literature (S17 Table). Reliable evidence from a robust meta-analysis of large randomised controlled trials has a 90% probability of high quality evidence with some uncertainty accruing from the presence of heterogeneity. Because only performance bias is realised, there is no downgrading for risk of bias (S17 Table).

In the same situation but with a few small trials (scenario two), the evidence becomes moderate to high as a result of imprecision (72% serious) and publication bias (28% suspected) accounting for the uncertainty inherent in meta-analyses of a few small studies.

For scenario three, the addition of known limitations (inadequate randomisation and surrogate outcomes) to the first scenario results in more severe downgrading (moderate to low overall) as a result of additional risk of bias (50% serious) and indirectness (70% serious to very serious).

Scenario four illustrates the accrual of probabilities across domains, where the probability of downgrading within a domain remains less than 50%. Thus the combination of potential problems is reflected in a high to moderate classification.

The final scenario represents low quality evidence where problems are more likely than not, across multiple domains, resulting in a 65% probability of very low evidence.

Agreement on overall reliability between the Bayesian network and the summary of findings in four reviews [27–30] assessing 13 outcomes was substantial (kappa 0.69; 95% confidence interval 0.38 to 0.99). There was one substantial discrepancy, where the original review did not downgrade evidence on the basis of imprecision despite the confidence intervals of the point estimate being consistent with benefit and harms. Minor discrepancies also occurred, most commonly in assessment of risk of bias, where standard assessment resulting in downgrading for the identification of a single problem (one instance) or where risk of bias was unclear (three instances). Reasons for these differences may relate to differing expertise or value judgements underlying GRADE assessment or judgments relating to the severity and relative weightings of domains. We emphasise that these are preliminary results intended as proof of principle. Additional work to validate the model more formally is presented in further work.

## Discussion

The GRADE framework provides a mechanism by which systematic review evidence can be interpreted in terms of quality of evidence to inform the development of guidelines or evidence briefings [2–16]. Despite providing a consistent framework for making transparent judgements, GRADE does not have high repeatability [3, 19]. The complexity of the approach, whilst necessary to allow nuanced judgements, also results in potential for misapplication, particularly with inexperienced reviewers or those lacking GRADE training. One approach to dealing with this is to ensure that all raters have sufficient experience and training. However, another approach is to see if statistical approaches can support novices based on the experience of others, including experienced raters. Users still require training to make consistent value judgements to parametise the model, but judgments regarding impact on overall validity are automated. Formal testing of consistency of application and congruence with existing mechanisms requires further exploration, but our initial results suggest that a quality assessment tool based on a probabilistic Bayesian network may prove a useful tool for GRADE implementation. We consider the potential advantages and limitations of probabilistic assessment below.

### Potential advantages of the tool

The primary advantages of the tool are that it requires users to consider all items and to consider them consistently. For example, sample size and number of studies have a bearing on our ability to detect both overall effects and specific biases, but the impacts are indirect and therefore not always considered. Thus a standard GRADE assessment applied by an inexperienced researcher may or may not distinguish between the results of robust, consistent meta-analyses based on large number of large studies or few small studies. Explicit inclusion of size and number of studies in the Bayesian network ensures appropriate circumspection in the latter circumstance, given that around a quarter of evidence from meta-analysis of few small trials is considered reliable [34].

Even where GRADE users consider all items, they may not coherently combine the information when making judgements. For example, high heterogeneity may be interpreted as inconsistency even when there is no variation in effect direction and the lower 95% confidence intervals of studies are consistent with a minimally important difference. The use of conditional probabilities ensures that these relationships are consistently and explicitly considered. This may be especially important when intermediate states are encountered. For example, it may be relatively easy to make a repeatable decision regarding imprecision when point estimates are consistent with benefits and harms, study numbers are low and sample sizes are small but if these items are intermediate, it is difficult to operationalize a consistent decision. The use of conditional probability tables mean that intermediate values can be calibrated with reference to worst and best case scenarios and applied consistently.

This current paper has shown that the tool largely reproduces GRADE judgements in a variety of scenarios which is consistent with other pilot evaluations of the tool. We have found evidence of moderate to substantial agreement between researchers’ responses to the checklist questions of the tool [17]. Similarly we have found moderate to substantial agreement on judgements for GRADE domains and overall quality ratings made by independent users of the tool [31] although further work is needed to improve agreement [31].

A less critical, but interesting property of the probabilistic assessment is the ability to accrue small probabilities across domains, resulting in accurate downgrading where multiple small but nonetheless important uncertainties exist. GRADE does allow uncertainties across multiple domains to be summed with a choice regarding the domain responsible for downgrading [2–16] but this is a pragmatic solution that can be hard to apply, particularly in a situation where there is a small probability of bias (say 0.2) in every domain. Rationally this should result in a downgrade from high to moderate quality evidence, but in practice users may operate using higher thresholds to signal concerns. For example, in our pilot evaluation comparing with standard GRADE assessments by an expert user we found a number of occasions where our tool was more conservative in judgements due to accumulation of probabilities across domains not taken into account in standard assessments [31].

In addition to providing a potential tool for the semi-automation of GRADE assessments, the Bayesian network may prove useful as a focus for methodological development. Some aspects of the GRADE framework are better supported by evidence than others. Changing the structure and parameterisation of the model allows exploration of the relative importance of different components. Methodological applications could involve mapping empirical probabilities and applying them or formally eliciting expert opinion to generate distributions for the probabilities, appropriately reflecting uncertainty. Other theoretical work could focus on the value of information associated with different problems, or relating quality of evidence to other aspects of research synthesis conduct via meta-epidemiology.

The impact of changes to the GRADE framework can be quantified and explored. For example, what is the impact of severe downgrading for moderate problems only when they are identified across five domains, rather than two? Probabilistic assessment could also facilitate comparison with other tools or allow adaptation for specific settings or other contexts such as syntheses of narrative, qualitative and non-randomised studies.

Probabilistic assessment has two other potentially desirable features. First, the reliability assessment can be represented as a probability distribution, allowing uncertainty to be expressed where appropriate. The approach also lends itself to automation, where nodes can be parameterised either using data mining software [32] or where frameworks such as ARCHIE (the Cochrane Collaboration's central server for managing documents) underpin review production and structure. Automation of research synthesis activities are receiving increasing attention [33], driven by increasing quantities of information and the need to inform ever more complex decisions with evidence efficiently. Probabilistic approaches may provide a future mechanism for automation of reliability assessment, which has hitherto been a missing component in proposed mechanisms for automated research synthesis.

### Potential limitations of the tool

A key strength of GRADE is that it forces users to make judgements about both the sources of bias and overall quality. The judgements may or may not be appropriate, but they are transparent. The Bayesian network uses the same logic in determining node states, for example forcing judgement that blinding of participants leads to bias or it does not. The subsequent impact of this decision is however, not left to the reviewers judgement. This is both strength and a weakness. The strength is that the impact of bias is consistent. The weakness is that the impact of blinding on risk of bias is dependent upon further context, which is unknown. In most circumstances, problems with blinding alone should not result in downgrading (which is how the Bayesian network is parameterised), but there are circumstances when the resulting performance bias may have serious or even very serious implications, downgrading high quality evidence. In theory, the factors which relate to the impact of blinding can be added to the Bayesian network, but in practice, beyond considering generic issues such as whether the outcome is objective, the network becomes overly complex for straightforward application. Further development of the evidence base underpinning the impact of biases may be required to address this issue adequately. Similar issues are raised when considering the impact of indirect comparisons given the diversity of views and embryonic evidence base regarding the relative merits of direct and indirect evidence in different contexts.

An uncertainty about our tool at present is the extent to which probabilities in our Bayesian network transfer across diverse topic areas. One of the advantages of our tool is that is that it provides a sound theoretical framework (through the use of Bayesian networks) to investigate these issues empirically in the future and as the evidence accumulates modifications to the model can be applied. Alternatively, there is sufficient flexibility in our approach that reviewers with belief or evidence that bias operates uniquely for a particular topic, outcome, or treatment comparison may modify the probabilities according to background knowledge as all inputs to the model have been made publically available in supplementary materials.

An advantage of our model is that it estimates the uncertainty in judgements on the strength of a body of evidence (e.g. 90% probability the evidence is low quality and 10% probability that it is moderate quality). In the present paper we haven’t determined specific thresholds for interpreting this uncertainty (e.g. if there is 90% probability of low quality evidence is that sufficient to conclude the body of evidence is low?). This is to allow flexibility for users and decision-makers to apply the tool in a way that reflects their needs and their context regarding interpretation of uncertainty. However, further work engaging with users and decision-makers applying the tool may be needed to identify whether determining more specific thresholds for interpretation would be beneficial. Thus, a collaborative GRADE assessment undertaken by experienced methodologists in conjunction with relevant subject experts is likely to yield a more nuanced validity assessment provided that the complexity of the decisions do not result in incoherence. It is also true that parameterisation of the Bayesian network requires a degree of training as it involves subjective value judgements notably regarding minimal important difference. Whilst it is possible to conceptualise a probabilistic model informed by systematically collated evidence or belief regarding these uncertainties, such a framework remains a distant reality. Ultimately the strengths and limitations of the Bayesian network and a standard approach to GRADE depend on the extent to which expert judgements are required to inform a validity assessment.

## Conclusions

Bayesian networks have considerable potential to facilitate probabilistic assessment of the reliability of research evidence. The key strength of such networks lies in the provision of a statistically coherent method for combining probabilities across a complex framework based on both belief and evidence. In addition to providing tools for less experienced users to implement reliability assessment, the potential for sensitivity analyses may be beneficial for the methodological development of reliability tools, and automation may also lead to efficiency gains.

## Supporting Information

S1 Checklist Questions for randomised meta-analysis strength of evidence assessment. (DOCX)

S1 Table Conditional probability table: Selection bias. (DOCX)

S2 Table Conditional probability table: Performance bias. (DOCX)

S3 Table Conditional probability table: Detection bias. (DOCX)

S4 Table Conditional probability table: Attrition bias. (DOCX)

S5 Table Conditional probability table: Reporting bias. (DOCX)

S6 Table Conditional probability table: Other bias. (DOCX)

S7 Table Conditional probability table: Risk of bias. (DOCX)

S8 Table Conditional probability table: Consistency. (DOCX)

S9 Table Conditional probability table: Strength of evidence. (DOCX)

S10 Table Conditional probability table: Study distribution. (DOCX)

S11 Table Conditional probability table: Inconsistency. (DOCX)

S12 Table Conditional probability table: Applicability. (DOCX)

S13 Table Conditional probability table: Indirectness. (DOCX)

S14 Table Conditional probability table: Statistical information. (DOCX)

S15 Table Conditional probability table: Imprecision. (DOCX)

S16 Table Conditional probability table: Publication bias. (DOCX)

S17 Table Probabilities of bias and overall reliability for five scenarios. (DOCX)
