# Supplementary material for: The Use of Bayesian Networks to Assess the Quality of Evidence from Research Synthesis: 1
Source: PLoS One. 2015 Apr 2;10(4):e0114497. doi: 10.1371/journal.pone.0114497 (PMC4383525; doi:10.1371/journal.pone.0114497)
Supplement: S1 Checklist — (DOCX) [file pone.0114497.s001.docx]

**Questions for randomised meta-analysis Strength of Evidence Tool**

**Risk of Bias**

1. Random sequence generation used? ( no potential for selection bias)

- Yes
- No
- Unclear

1. Allocation concealment used? (no potential for selection bias)

- Yes
- No
- Unclear

1. Blinding of participants and personnel? (no potential for performance bias)

- Yes
- No
- Unclear

1. Blinding of outcome assessment? (no potential for detection bias)

- Yes
- No
- Unclear

1. Objective outcome used?

- Yes
- No

1. More than 80% of participants enrolled in trials included in the analysis?

- Yes
- No
- Unclear

1. Data reported consistently for the outcome of interest? (no potential selective reporting)

- Yes
- No
- Unclear

1. No other biases reported? (no potential of other bias)

- Yes
- No

1. Trial ended as scheduled? (not stopped early)

- Yes
- No

**Inconsistency**

1. Point estimates consistent?

- Yes
- No

1. Extent of confidence interval overlap

- Substantial overlap

(all confidence intervals overlap at least one of the included studies point estimate)

- Some overlap

(some but not all confidence intervals overlap at least one point estimate)

- No overlap

(at least one outlier (confidence intervals for one or more studies do not overlap with most other included studies)

1. Direction of effect consistent?

- Yes
- No

1. Magnitude of statistical heterogeneity? (measured by I^2^)

- Low (I^2^ <40%)
- Moderate (I^2^ 40% to 60%)
- High (I^2^>60%)

1. Test for heterogeneity statistically significant? (p<0.1)

- Not statistically significant
- Statistically significant

**Indirectness**

1. Populations in included studies applicable to the decision context?

- Highly applicable
- Applicable
- Poorly applicable

1. Interventions and comparators in the included studies applicable to the decision context?

- Highly applicable
- Applicable
- Poorly applicable

1. The included outcome is a clinically meaningful endpoint (i.e. not a surrogate)?

- Yes
- No

1. Outcome timeframe sufficient and measured outcome reflects intended outcome?

- Sufficient/yes
- Insufficient/no

1. Conclusions of the review based on direct comparisons?

- Yes
- No

**Imprecision**

1. Was the pooled confidence interval sufficiently narrow? (i.e. pooled estimate not consistent with benefit and harm)

- Yes
- No

1. Sample size for most studies?

- High (> 300 participants)
- Intermediate (100 to 300 participants)
- Low (<100 participants)

1. Number of included studies?

- Large (10 studies)
- Moderate (5 to 10 studies)
- Small (<5 studies)

1. Outcome a common event? (occurs more than 1/100)

- Yes
- No
- Not applicable (not a dichotomous outcome)

1. No evidence of serious harm associated with treatment?

- Yes
- No

**Publication Bias**

1. Did the authors conduct a comprehensive search?

- Yes
- No

1. Did the authors search for grey literature?

- Yes
- No

1. Were language restrictions applied to study selection ?

- Yes
- No

1. Was there industry influence on studies included in the review?

- Yes
- No

1. Evidence of funnel plot asymmetry?

- Yes
- No
- Unclear

1. Discrepancy in findings between published and unpublished trials?

- Yes
- No
- Unclear
