# Supplementary material for: The Use of Bayesian Networks to Assess the Quality of Evidence from Research Synthesis: 1
Source: PLoS One. 2015 Apr 2;10(4):e0114497. doi: 10.1371/journal.pone.0114497 (PMC4383525; doi:10.1371/journal.pone.0114497)
Supplement: S1 Table — (DOCX) [file pone.0114497.s002.docx]

| Random sequence generation | **yes** | | | **no** | | | **unclear** | | |
| --- | --- | --- | --- | --- | --- | --- | --- | --- | --- |
| Allocation concealment | **yes** | **no** | **unclear** | **yes** | **no** | **unclear** | **yes** | **no** | **unclear** |
| high | 0 | 0.8 | 0 | 0.8 | 1 | 0.7 | 0 | 0.7 | 0 |
| low | 1 | 0.2 | 0.7 | 0.2 | 0 | 0 | 0.7 | 0 | 0 |
| unclear | 0 | 0 | 0.3 | 0 | 0 | 0.3 | 0.3 | 0.3 | 1 |

Table S1. Conditional probability table: Selection bias
