# Supplementary material for: The Use of Bayesian Networks to Assess the Quality of Evidence from Research Synthesis: 1
Source: PLoS One. 2015 Apr 2;10(4):e0114497. doi: 10.1371/journal.pone.0114497 (PMC4383525; doi:10.1371/journal.pone.0114497)
Supplement: S3 Table — (DOCX) [file pone.0114497.s004.docx]

| Blinding of outcome assessor | **yes** | | **no** | | **unclear** | |
| --- | --- | --- | --- | --- | --- | --- |
| Objective outcome | **yes** | **no** | **yes** | **no** | **yes** | **no** |
| high | 0 | 0 | 0 | 1 | 0 | 0.33 |
| low | 1 | 1 | 1 | 0 | 1 | 0.33 |
| unclear | 0 | 0 | 0 | 0 | 0 | 0.34 |

Table S3. Conditional probability table: Detection bias
