# Supplementary material for: The Use of Bayesian Networks to Assess the Quality of Evidence from Research Synthesis: 1
Source: PLoS One. 2015 Apr 2;10(4):e0114497. doi: 10.1371/journal.pone.0114497 (PMC4383525; doi:10.1371/journal.pone.0114497)
Supplement: S4 Table — (DOCX) [file pone.0114497.s005.docx]

| Complete outcome data | no | yes | unclear |
| --- | --- | --- | --- |
| high | 1 | 0 | 0 |
| low | 0 | 1 | 0 |
| unclear | 0 | 0 | 1 |

Table S4. Conditional probability table: Attrition bias
