# Supplementary material for: The Use of Bayesian Networks to Assess the Quality of Evidence from Research Synthesis: 1
Source: PLoS One. 2015 Apr 2;10(4):e0114497. doi: 10.1371/journal.pone.0114497 (PMC4383525; doi:10.1371/journal.pone.0114497)
Supplement: S6 Table — (DOCX) [file pone.0114497.s007.docx]

| No stopping early | **no** | | **yes** | |
| --- | --- | --- | --- | --- |
| No other bias | **no** | **yes** | **no** | **yes** |
| high | 1 | 0.5 | 0.5 | 0 |
| low | 0 | 0.5 | 0.5 | 1 |
| unclear | 1 | 0.5 | 0.5 | 0 |

Table S6. Conditional probability table: Other bias
