# Supplementary material for: The Use of Bayesian Networks to Assess the Quality of Evidence from Research Synthesis: 1
Source: PLoS One. 2015 Apr 2;10(4):e0114497. doi: 10.1371/journal.pone.0114497 (PMC4383525; doi:10.1371/journal.pone.0114497)
Supplement: S7 Table — (DOCX) [file pone.0114497.s008.docx]

| Selection bias | Performance bias | Detection bias | Attrition bias | Reporting bias | Other | no | serious | very serious |
| --- | --- | --- | --- | --- | --- | --- | --- | --- |
| high | high | high | high | high | high | 0 | 0 | 1 |
| high | low | high | high | high | high | 0 | 0 | 1 |
| high | unclear | high | high | high | high | 0 | 0 | 1 |
| high | high | low | high | high | high | 0 | 0 | 1 |
| high | low | low | high | high | high | 0 | 0 | 1 |
| high | unclear | low | high | high | high | 0 | 0 | 1 |
| high | high | unclear | high | high | high | 0 | 0 | 1 |
| high | low | unclear | high | high | high | 0 | 0 | 1 |
| high | unclear | unclear | high | high | high | 0 | 0 | 1 |
| high | high | high | low | high | high | 0 | 0 | 1 |
| high | low | high | low | high | high | 0 | 0 | 1 |
| high | unclear | high | low | high | high | 0 | 0 | 1 |
| high | high | low | low | high | high | 0 | 0 | 1 |
| high | low | low | low | high | high | 0 | 0 | 1 |
| high | unclear | low | low | high | high | 0 | 0 | 1 |
| high | high | unclear | low | high | high | 0 | 0 | 1 |
| high | low | unclear | low | high | high | 0 | 0 | 1 |
| high | unclear | unclear | low | high | high | 0 | 0 | 1 |
| high | high | high | unclear | high | high | 0 | 0 | 1 |
| high | low | high | unclear | high | high | 0 | 0 | 1 |
| high | unclear | high | unclear | high | high | 0 | 0 | 1 |
| high | high | low | unclear | high | high | 0 | 0 | 1 |
| high | low | low | unclear | high | high | 0 | 0 | 1 |
| high | unclear | low | unclear | high | high | 0 | 0 | 1 |
| high | high | unclear | unclear | high | high | 0 | 0 | 1 |
| high | low | unclear | unclear | high | high | 0 | 0 | 1 |
| high | unclear | unclear | unclear | high | high | 0 | 0 | 1 |
| high | high | high | high | low | high | 0 | 0 | 1 |
| high | low | high | high | low | high | 0 | 0 | 1 |
| high | unclear | high | high | low | high | 0 | 0 | 1 |
| high | high | low | high | low | high | 0 | 0 | 1 |
| high | low | low | high | low | high | 0 | 0 | 1 |
| high | unclear | low | high | low | high | 0 | 0 | 1 |
| high | high | unclear | high | low | high | 0 | 0 | 1 |
| high | low | unclear | high | low | high | 0 | 0 | 1 |
| high | unclear | unclear | high | low | high | 0 | 0 | 1 |
| high | high | high | low | low | high | 0 | 0 | 1 |
| high | low | high | low | low | high | 0 | 0 | 1 |
| high | unclear | high | low | low | high | 0 | 0 | 1 |
| high | high | low | low | low | high | 0 | 0 | 1 |
| high | low | low | low | low | high | 0 | 0 | 1 |
| high | unclear | low | low | low | high | 0 | 0 | 1 |
| high | high | unclear | low | low | high | 0 | 0 | 1 |
| high | low | unclear | low | low | high | 0 | 0 | 1 |
| high | unclear | unclear | low | low | high | 0 | 0 | 1 |
| high | high | high | unclear | low | high | 0 | 0 | 1 |
| high | low | high | unclear | low | high | 0 | 0 | 1 |
| high | unclear | high | unclear | low | high | 0 | 0 | 1 |
| high | high | low | unclear | low | high | 0 | 0 | 1 |
| high | low | low | unclear | low | high | 0 | 0 | 1 |
| high | unclear | low | unclear | low | high | 0 | 0 | 1 |
| high | high | unclear | unclear | low | high | 0 | 0 | 1 |
| high | low | unclear | unclear | low | high | 0 | 0 | 1 |
| high | unclear | unclear | unclear | low | high | 0 | 0 | 1 |
| high | high | high | high | unclear | high | 0 | 0 | 1 |
| high | low | high | high | unclear | high | 0 | 0 | 1 |
| high | unclear | high | high | unclear | high | 0 | 0 | 1 |
| high | high | low | high | unclear | high | 0 | 0 | 1 |
| high | low | low | high | unclear | high | 0 | 0 | 1 |
| high | unclear | low | high | unclear | high | 0 | 0 | 1 |
| high | high | unclear | high | unclear | high | 0 | 0 | 1 |
| high | low | unclear | high | unclear | high | 0 | 0 | 1 |
| high | unclear | unclear | high | unclear | high | 0 | 0 | 1 |
| high | high | high | low | unclear | high | 0 | 0 | 1 |
| high | low | high | low | unclear | high | 0 | 0 | 1 |
| high | unclear | high | low | unclear | high | 0 | 0 | 1 |
| high | high | low | low | unclear | high | 0 | 0 | 1 |
| high | low | low | low | unclear | high | 0 | 0 | 1 |
| high | unclear | low | low | unclear | high | 0 | 0 | 1 |
| high | high | unclear | low | unclear | high | 0 | 0 | 1 |
| high | low | unclear | low | unclear | high | 0 | 0 | 1 |
| high | unclear | unclear | low | unclear | high | 0 | 0 | 1 |
| high | high | high | unclear | unclear | high | 0 | 0 | 1 |
| high | low | high | unclear | unclear | high | 0 | 0 | 1 |
| high | unclear | high | unclear | unclear | high | 0 | 0 | 1 |
| high | high | low | unclear | unclear | high | 0 | 0 | 1 |
| high | low | low | unclear | unclear | high | 0 | 0 | 1 |
| high | unclear | low | unclear | unclear | high | 0 | 0 | 1 |
| high | high | unclear | unclear | unclear | high | 0 | 0 | 1 |
| high | low | unclear | unclear | unclear | high | 0 | 0 | 1 |
| high | unclear | unclear | unclear | unclear | high | 0 | 0 | 1 |
| high | high | high | high | high | low | 0 | 0 | 1 |
| high | low | high | high | high | low | 0 | 0 | 1 |
| high | unclear | high | high | high | low | 0 | 0 | 1 |
| high | high | low | high | high | low | 0 | 0 | 1 |
| high | low | low | high | high | low | 0 | 0 | 1 |
| high | unclear | low | high | high | low | 0 | 0 | 1 |
| high | high | unclear | high | high | low | 0 | 0 | 1 |
| high | low | unclear | high | high | low | 0 | 0 | 1 |
| high | unclear | unclear | high | high | low | 0 | 0 | 1 |
| high | high | high | low | high | low | 0 | 0 | 1 |
| high | low | high | low | high | low | 0 | 0 | 1 |
| high | unclear | high | low | high | low | 0 | 0 | 1 |
| high | high | low | low | high | low | 0 | 0 | 1 |
| high | low | low | low | high | low | 0 | 0 | 1 |
| high | unclear | low | low | high | low | 0 | 0 | 1 |
| high | high | unclear | low | high | low | 0 | 0 | 1 |
| high | low | unclear | low | high | low | 0 | 0 | 1 |
| high | unclear | unclear | low | high | low | 0 | 0 | 1 |
| high | high | high | unclear | high | low | 0 | 0 | 1 |
| high | low | high | unclear | high | low | 0 | 0 | 1 |
| high | unclear | high | unclear | high | low | 0 | 0 | 1 |
| high | high | low | unclear | high | low | 0 | 0 | 1 |
| high | low | low | unclear | high | low | 0 | 0 | 1 |
| high | unclear | low | unclear | high | low | 0 | 0 | 1 |
| high | high | unclear | unclear | high | low | 0 | 0 | 1 |
| high | low | unclear | unclear | high | low | 0 | 0 | 1 |
| high | unclear | unclear | unclear | high | low | 0 | 0 | 1 |
| high | high | high | high | low | low | 0 | 0 | 1 |
| high | low | high | high | low | low | 0 | 0 | 1 |
| high | unclear | high | high | low | low | 0 | 0 | 1 |
| high | high | low | high | low | low | 0 | 0 | 1 |
| high | low | low | high | low | low | 0 | 0 | 1 |
| high | unclear | low | high | low | low | 0 | 0 | 1 |
| high | high | unclear | high | low | low | 0 | 0 | 1 |
| high | low | unclear | high | low | low | 0 | 0 | 1 |
| high | unclear | unclear | high | low | low | 0 | 0 | 1 |
| high | high | high | low | low | low | 0 | 0 | 1 |
| high | low | high | low | low | low | 0 | 0 | 1 |
| high | unclear | high | low | low | low | 0 | 0 | 1 |
| high | high | low | low | low | low | 0 | 0 | 1 |
| high | low | low | low | low | low | 0 | 1 | 0 |
| high | unclear | low | low | low | low | 0 | 1 | 0 |
| high | high | unclear | low | low | low | 0 | 0 | 1 |
| high | low | unclear | low | low | low | 0 | 1 | 0 |
| high | unclear | unclear | low | low | low | 0 | 1 | 0 |
| high | high | high | unclear | low | low | 0 | 0 | 1 |
| high | low | high | unclear | low | low | 0 | 0 | 1 |
| high | unclear | high | unclear | low | low | 0 | 0 | 1 |
| high | high | low | unclear | low | low | 0 | 0 | 1 |
| high | low | low | unclear | low | low | 0 | 1 | 0 |
| high | unclear | low | unclear | low | low | 0 | 1 | 0 |
| high | high | unclear | unclear | low | low | 0 | 0 | 1 |
| high | low | unclear | unclear | low | low | 0 | 1 | 0 |
| high | unclear | unclear | unclear | low | low | 0 | 1 | 0 |
| high | high | high | high | unclear | low | 0 | 0 | 1 |
| high | low | high | high | unclear | low | 0 | 0 | 1 |
| high | unclear | high | high | unclear | low | 0 | 0 | 1 |
| high | high | low | high | unclear | low | 0 | 0 | 1 |
| high | low | low | high | unclear | low | 0 | 0 | 1 |
| high | unclear | low | high | unclear | low | 0 | 0 | 1 |
| high | high | unclear | high | unclear | low | 0 | 0 | 1 |
| high | low | unclear | high | unclear | low | 0 | 0 | 1 |
| high | unclear | unclear | high | unclear | low | 0 | 0 | 1 |
| high | high | high | low | unclear | low | 0 | 0 | 1 |
| high | low | high | low | unclear | low | 0 | 0 | 1 |
| high | unclear | high | low | unclear | low | 0 | 0 | 1 |
| high | high | low | low | unclear | low | 0 | 0 | 1 |
| high | low | low | low | unclear | low | 0 | 1 | 0 |
| high | unclear | low | low | unclear | low | 0 | 1 | 0 |
| high | high | unclear | low | unclear | low | 0 | 0 | 1 |
| high | low | unclear | low | unclear | low | 0 | 1 | 0 |
| high | unclear | unclear | low | unclear | low | 0 | 1 | 0 |
| high | high | high | unclear | unclear | low | 0 | 0 | 1 |
| high | low | high | unclear | unclear | low | 0 | 0 | 1 |
| high | unclear | high | unclear | unclear | low | 0 | 0 | 1 |
| high | high | low | unclear | unclear | low | 0 | 0 | 1 |
| high | low | low | unclear | unclear | low | 0 | 1 | 0 |
| high | unclear | low | unclear | unclear | low | 0 | 1 | 0 |
| high | high | unclear | unclear | unclear | low | 0 | 0 | 1 |
| high | low | unclear | unclear | unclear | low | 0 | 1 | 0 |
| high | unclear | unclear | unclear | unclear | low | 0 | 1 | 0 |
| low | high | high | high | high | high | 0 | 0 | 1 |
| low | low | high | high | high | high | 0 | 0 | 1 |
| low | unclear | high | high | high | high | 0 | 0 | 1 |
| low | high | low | high | high | high | 0 | 0 | 1 |
| low | low | low | high | high | high | 0 | 0 | 1 |
| low | unclear | low | high | high | high | 0 | 0 | 1 |
| low | high | unclear | high | high | high | 0 | 0 | 1 |
| low | low | unclear | high | high | high | 0 | 0 | 1 |
| low | unclear | unclear | high | high | high | 0 | 0 | 1 |
| low | high | high | low | high | high | 0 | 0 | 1 |
| low | low | high | low | high | high | 0 | 0 | 1 |
| low | unclear | high | low | high | high | 0 | 0 | 1 |
| low | high | low | low | high | high | 0 | 0 | 1 |
| low | low | low | low | high | high | 0 | 1 | 0 |
| low | unclear | low | low | high | high | 0 | 1 | 0 |
| low | high | unclear | low | high | high | 0 | 0 | 1 |
| low | low | unclear | low | high | high | 0 | 1 | 0 |
| low | unclear | unclear | low | high | high | 0 | 1 | 0 |
| low | high | high | unclear | high | high | 0 | 0 | 1 |
| low | low | high | unclear | high | high | 0 | 0 | 1 |
| low | unclear | high | unclear | high | high | 0 | 0 | 1 |
| low | high | low | unclear | high | high | 0 | 0 | 1 |
| low | low | low | unclear | high | high | 0 | 1 | 0 |
| low | unclear | low | unclear | high | high | 0 | 1 | 0 |
| low | high | unclear | unclear | high | high | 0 | 0 | 1 |
| low | low | unclear | unclear | high | high | 0 | 1 | 0 |
| low | unclear | unclear | unclear | high | high | 0 | 1 | 0 |
| low | high | high | high | low | high | 0 | 0 | 1 |
| low | low | high | high | low | high | 0 | 0 | 1 |
| low | unclear | high | high | low | high | 0 | 0 | 1 |
| low | high | low | high | low | high | 0 | 0 | 1 |
| low | low | low | high | low | high | 0 | 1 | 0 |
| low | unclear | low | high | low | high | 0 | 1 | 0 |
| low | high | unclear | high | low | high | 0 | 0 | 1 |
| low | low | unclear | high | low | high | 0 | 1 | 0 |
| low | unclear | unclear | high | low | high | 0 | 1 | 0 |
| low | high | high | low | low | high | 0 | 0 | 1 |
| low | low | high | low | low | high | 0 | 1 | 0 |
| low | unclear | high | low | low | high | 0 | 1 | 0 |
| low | high | low | low | low | high | 0 | 1 | 0 |
| low | low | low | low | low | high | 1 | 0 | 0 |
| low | unclear | low | low | low | high | 1 | 0 | 0 |
| low | high | unclear | low | low | high | 0 | 1 | 0 |
| low | low | unclear | low | low | high | 1 | 0 | 0 |
| low | unclear | unclear | low | low | high | 1 | 0 | 0 |
| low | high | high | unclear | low | high | 0 | 0 | 1 |
| low | low | high | unclear | low | high | 0 | 1 | 0 |
| low | unclear | high | unclear | low | high | 0 | 1 | 0 |
| low | high | low | unclear | low | high | 0 | 1 | 0 |
| low | low | low | unclear | low | high | 1 | 0 | 0 |
| low | unclear | low | unclear | low | high | 1 | 0 | 0 |
| low | high | unclear | unclear | low | high | 0 | 1 | 0 |
| low | low | unclear | unclear | low | high | 1 | 0 | 0 |
| low | unclear | unclear | unclear | low | high | 1 | 0 | 0 |
| low | high | high | high | unclear | high | 0 | 0 | 1 |
| low | low | high | high | unclear | high | 0 | 0 | 1 |
| low | unclear | high | high | unclear | high | 0 | 0 | 1 |
| low | high | low | high | unclear | high | 0 | 0 | 1 |
| low | low | low | high | unclear | high | 0 | 1 | 0 |
| low | unclear | low | high | unclear | high | 0 | 1 | 0 |
| low | high | unclear | high | unclear | high | 0 | 0 | 1 |
| low | low | unclear | high | unclear | high | 0 | 1 | 0 |
| low | unclear | unclear | high | unclear | high | 0 | 1 | 0 |
| low | high | high | low | unclear | high | 0 | 0 | 1 |
| low | low | high | low | unclear | high | 0 | 1 | 0 |
| low | unclear | high | low | unclear | high | 0 | 1 | 0 |
| low | high | low | low | unclear | high | 0 | 1 | 0 |
| low | low | low | low | unclear | high | 1 | 0 | 0 |
| low | unclear | low | low | unclear | high | 1 | 0 | 0 |
| low | high | unclear | low | unclear | high | 0 | 1 | 0 |
| low | low | unclear | low | unclear | high | 1 | 0 | 0 |
| low | unclear | unclear | low | unclear | high | 1 | 0 | 0 |
| low | high | high | unclear | unclear | high | 0 | 0 | 1 |
| low | low | high | unclear | unclear | high | 0 | 1 | 0 |
| low | unclear | high | unclear | unclear | high | 0 | 1 | 0 |
| low | high | low | unclear | unclear | high | 0 | 1 | 0 |
| low | low | low | unclear | unclear | high | 1 | 0 | 0 |
| low | unclear | low | unclear | unclear | high | 1 | 0 | 0 |
| low | high | unclear | unclear | unclear | high | 0 | 1 | 0 |
| low | low | unclear | unclear | unclear | high | 1 | 0 | 0 |
| low | unclear | unclear | unclear | unclear | high | 1 | 0 | 0 |
| low | high | high | high | high | low | 0 | 0 | 1 |
| low | low | high | high | high | low | 0 | 0 | 1 |
| low | unclear | high | high | high | low | 0 | 0 | 1 |
| low | high | low | high | high | low | 0 | 0 | 1 |
| low | low | low | high | high | low | 0 | 1 | 0 |
| low | unclear | low | high | high | low | 0 | 1 | 0 |
| low | high | unclear | high | high | low | 0 | 0 | 1 |
| low | low | unclear | high | high | low | 0 | 1 | 0 |
| low | unclear | unclear | high | high | low | 0 | 1 | 0 |
| low | high | high | low | high | low | 0 | 0 | 1 |
| low | low | high | low | high | low | 0 | 1 | 0 |
| low | unclear | high | low | high | low | 0 | 1 | 0 |
| low | high | low | low | high | low | 0 | 1 | 0 |
| low | low | low | low | high | low | 1 | 0 | 0 |
| low | unclear | low | low | high | low | 1 | 0 | 0 |
| low | high | unclear | low | high | low | 0 | 1 | 0 |
| low | low | unclear | low | high | low | 1 | 0 | 0 |
| low | unclear | unclear | low | high | low | 1 | 0 | 0 |
| low | high | high | unclear | high | low | 0 | 0 | 1 |
| low | low | high | unclear | high | low | 0 | 1 | 0 |
| low | unclear | high | unclear | high | low | 0 | 1 | 0 |
| low | high | low | unclear | high | low | 0 | 1 | 0 |
| low | low | low | unclear | high | low | 1 | 0 | 0 |
| low | unclear | low | unclear | high | low | 1 | 0 | 0 |
| low | high | unclear | unclear | high | low | 0 | 1 | 0 |
| low | low | unclear | unclear | high | low | 1 | 0 | 0 |
| low | unclear | unclear | unclear | high | low | 1 | 0 | 0 |
| low | high | high | high | low | low | 0 | 0 | 1 |
| low | low | high | high | low | low | 0 | 1 | 0 |
| low | unclear | high | high | low | low | 0 | 1 | 0 |
| low | high | low | high | low | low | 0 | 1 | 0 |
| low | low | low | high | low | low | 1 | 0 | 0 |
| low | unclear | low | high | low | low | 1 | 0 | 0 |
| low | high | unclear | high | low | low | 0 | 1 | 0 |
| low | low | unclear | high | low | low | 1 | 0 | 0 |
| low | unclear | unclear | high | low | low | 1 | 0 | 0 |
| low | high | high | low | low | low | 0 | 1 | 0 |
| low | low | high | low | low | low | 1 | 0 | 0 |
| low | unclear | high | low | low | low | 1 | 0 | 0 |
| low | high | low | low | low | low | 1 | 0 | 0 |
| low | low | low | low | low | low | 1 | 0 | 0 |
| low | unclear | low | low | low | low | 1 | 0 | 0 |
| low | high | unclear | low | low | low | 1 | 0 | 0 |
| low | low | unclear | low | low | low | 1 | 0 | 0 |
| low | unclear | unclear | low | low | low | 1 | 0 | 0 |
| low | high | high | unclear | low | low | 0 | 1 | 0 |
| low | low | high | unclear | low | low | 1 | 0 | 0 |
| low | unclear | high | unclear | low | low | 1 | 0 | 0 |
| low | high | low | unclear | low | low | 1 | 0 | 0 |
| low | low | low | unclear | low | low | 1 | 0 | 0 |
| low | unclear | low | unclear | low | low | 1 | 0 | 0 |
| low | high | unclear | unclear | low | low | 1 | 0 | 0 |
| low | low | unclear | unclear | low | low | 1 | 0 | 0 |
| low | unclear | unclear | unclear | low | low | 1 | 0 | 0 |
| low | high | high | high | unclear | low | 0 | 0 | 1 |
| low | low | high | high | unclear | low | 0 | 1 | 0 |
| low | unclear | high | high | unclear | low | 0 | 1 | 0 |
| low | high | low | high | unclear | low | 0 | 1 | 0 |
| low | low | low | high | unclear | low | 1 | 0 | 0 |
| low | unclear | low | high | unclear | low | 1 | 0 | 0 |
| low | high | unclear | high | unclear | low | 0 | 1 | 0 |
| low | low | unclear | high | unclear | low | 1 | 0 | 0 |
| low | unclear | unclear | high | unclear | low | 1 | 0 | 0 |
| low | high | high | low | unclear | low | 0 | 1 | 0 |
| low | low | high | low | unclear | low | 1 | 0 | 0 |
| low | unclear | high | low | unclear | low | 1 | 0 | 0 |
| low | high | low | low | unclear | low | 1 | 0 | 0 |
| low | low | low | low | unclear | low | 1 | 0 | 0 |
| low | unclear | low | low | unclear | low | 1 | 0 | 0 |
| low | high | unclear | low | unclear | low | 1 | 0 | 0 |
| low | low | unclear | low | unclear | low | 1 | 0 | 0 |
| low | unclear | unclear | low | unclear | low | 1 | 0 | 0 |
| low | high | high | unclear | unclear | low | 0 | 1 | 0 |
| low | low | high | unclear | unclear | low | 1 | 0 | 0 |
| low | unclear | high | unclear | unclear | low | 1 | 0 | 0 |
| low | high | low | unclear | unclear | low | 1 | 0 | 0 |
| low | low | low | unclear | unclear | low | 1 | 0 | 0 |
| low | unclear | low | unclear | unclear | low | 1 | 0 | 0 |
| low | high | unclear | unclear | unclear | low | 1 | 0 | 0 |
| low | low | unclear | unclear | unclear | low | 1 | 0 | 0 |
| low | unclear | unclear | unclear | unclear | low | 1 | 0 | 0 |
| unclear | high | high | high | high | high | 0 | 0 | 1 |
| unclear | low | high | high | high | high | 0 | 0 | 1 |
| unclear | unclear | high | high | high | high | 0 | 0 | 1 |
| unclear | high | low | high | high | high | 0 | 0 | 1 |
| unclear | low | low | high | high | high | 0 | 0 | 1 |
| unclear | unclear | low | high | high | high | 0 | 0 | 1 |
| unclear | high | unclear | high | high | high | 0 | 0 | 1 |
| unclear | low | unclear | high | high | high | 0 | 0 | 1 |
| unclear | unclear | unclear | high | high | high | 0 | 0 | 1 |
| unclear | high | high | low | high | high | 0 | 0 | 1 |
| unclear | low | high | low | high | high | 0 | 0 | 1 |
| unclear | unclear | high | low | high | high | 0 | 0 | 1 |
| unclear | high | low | low | high | high | 0 | 0 | 1 |
| unclear | low | low | low | high | high | 0 | 1 | 0 |
| unclear | unclear | low | low | high | high | 0 | 1 | 0 |
| unclear | high | unclear | low | high | high | 0 | 0 | 1 |
| unclear | low | unclear | low | high | high | 0 | 1 | 0 |
| unclear | unclear | unclear | low | high | high | 0 | 1 | 0 |
| unclear | high | high | unclear | high | high | 0 | 0 | 1 |
| unclear | low | high | unclear | high | high | 0 | 0 | 1 |
| unclear | unclear | high | unclear | high | high | 0 | 0 | 1 |
| unclear | high | low | unclear | high | high | 0 | 0 | 1 |
| unclear | low | low | unclear | high | high | 0 | 1 | 0 |
| unclear | unclear | low | unclear | high | high | 0 | 1 | 0 |
| unclear | high | unclear | unclear | high | high | 0 | 0 | 1 |
| unclear | low | unclear | unclear | high | high | 0 | 1 | 0 |
| unclear | unclear | unclear | unclear | high | high | 0 | 1 | 0 |
| unclear | high | high | high | low | high | 0 | 0 | 1 |
| unclear | low | high | high | low | high | 0 | 0 | 1 |
| unclear | unclear | high | high | low | high | 0 | 0 | 1 |
| unclear | high | low | high | low | high | 0 | 0 | 1 |
| unclear | low | low | high | low | high | 0 | 1 | 0 |
| unclear | unclear | low | high | low | high | 0 | 1 | 0 |
| unclear | high | unclear | high | low | high | 0 | 0 | 1 |
| unclear | low | unclear | high | low | high | 0 | 1 | 0 |
| unclear | unclear | unclear | high | low | high | 0 | 1 | 0 |
| unclear | high | high | low | low | high | 0 | 0 | 1 |
| unclear | low | high | low | low | high | 0 | 1 | 0 |
| unclear | unclear | high | low | low | high | 0 | 1 | 0 |
| unclear | high | low | low | low | high | 0 | 1 | 0 |
| unclear | low | low | low | low | high | 1 | 0 | 0 |
| unclear | unclear | low | low | low | high | 1 | 0 | 0 |
| unclear | high | unclear | low | low | high | 0 | 1 | 0 |
| unclear | low | unclear | low | low | high | 1 | 0 | 0 |
| unclear | unclear | unclear | low | low | high | 1 | 0 | 0 |
| unclear | high | high | unclear | low | high | 0 | 0 | 1 |
| unclear | low | high | unclear | low | high | 0 | 1 | 0 |
| unclear | unclear | high | unclear | low | high | 0 | 1 | 0 |
| unclear | high | low | unclear | low | high | 0 | 1 | 0 |
| unclear | low | low | unclear | low | high | 1 | 0 | 0 |
| unclear | unclear | low | unclear | low | high | 1 | 0 | 0 |
| unclear | high | unclear | unclear | low | high | 0 | 1 | 0 |
| unclear | low | unclear | unclear | low | high | 1 | 0 | 0 |
| unclear | unclear | unclear | unclear | low | high | 1 | 0 | 0 |
| unclear | high | high | high | unclear | high | 0 | 0 | 1 |
| unclear | low | high | high | unclear | high | 0 | 0 | 1 |
| unclear | unclear | high | high | unclear | high | 0 | 0 | 1 |
| unclear | high | low | high | unclear | high | 0 | 0 | 1 |
| unclear | low | low | high | unclear | high | 0 | 1 | 0 |
| unclear | unclear | low | high | unclear | high | 0 | 1 | 0 |
| unclear | high | unclear | high | unclear | high | 0 | 0 | 1 |
| unclear | low | unclear | high | unclear | high | 0 | 1 | 0 |
| unclear | unclear | unclear | high | unclear | high | 0 | 1 | 0 |
| unclear | high | high | low | unclear | high | 0 | 0 | 1 |
| unclear | low | high | low | unclear | high | 0 | 1 | 0 |
| unclear | unclear | high | low | unclear | high | 0 | 1 | 0 |
| unclear | high | low | low | unclear | high | 0 | 1 | 0 |
| unclear | low | low | low | unclear | high | 1 | 0 | 0 |
| unclear | unclear | low | low | unclear | high | 1 | 0 | 0 |
| unclear | high | unclear | low | unclear | high | 0 | 1 | 0 |
| unclear | low | unclear | low | unclear | high | 1 | 0 | 0 |
| unclear | unclear | unclear | low | unclear | high | 1 | 0 | 0 |
| unclear | high | high | unclear | unclear | high | 0 | 0 | 1 |
| unclear | low | high | unclear | unclear | high | 0 | 1 | 0 |
| unclear | unclear | high | unclear | unclear | high | 0 | 1 | 0 |
| unclear | high | low | unclear | unclear | high | 0 | 1 | 0 |
| unclear | low | low | unclear | unclear | high | 1 | 0 | 0 |
| unclear | unclear | low | unclear | unclear | high | 1 | 0 | 0 |
| unclear | high | unclear | unclear | unclear | high | 0 | 1 | 0 |
| unclear | low | unclear | unclear | unclear | high | 1 | 0 | 0 |
| unclear | unclear | unclear | unclear | unclear | high | 1 | 0 | 0 |
| unclear | high | high | high | high | low | 0 | 0 | 1 |
| unclear | low | high | high | high | low | 0 | 0 | 1 |
| unclear | unclear | high | high | high | low | 0 | 0 | 1 |
| unclear | high | low | high | high | low | 0 | 0 | 1 |
| unclear | low | low | high | high | low | 0 | 1 | 0 |
| unclear | unclear | low | high | high | low | 0 | 1 | 0 |
| unclear | high | unclear | high | high | low | 0 | 0 | 1 |
| unclear | low | unclear | high | high | low | 0 | 1 | 0 |
| unclear | unclear | unclear | high | high | low | 0 | 1 | 0 |
| unclear | high | high | low | high | low | 0 | 0 | 1 |
| unclear | low | high | low | high | low | 0 | 1 | 0 |
| unclear | unclear | high | low | high | low | 0 | 1 | 0 |
| unclear | high | low | low | high | low | 0 | 1 | 0 |
| unclear | low | low | low | high | low | 1 | 0 | 0 |
| unclear | unclear | low | low | high | low | 1 | 0 | 0 |
| unclear | high | unclear | low | high | low | 0 | 1 | 0 |
| unclear | low | unclear | low | high | low | 1 | 0 | 0 |
| unclear | unclear | unclear | low | high | low | 1 | 0 | 0 |
| unclear | high | high | unclear | high | low | 0 | 0 | 1 |
| unclear | low | high | unclear | high | low | 0 | 1 | 0 |
| unclear | unclear | high | unclear | high | low | 0 | 1 | 0 |
| unclear | high | low | unclear | high | low | 0 | 1 | 0 |
| unclear | low | low | unclear | high | low | 1 | 0 | 0 |
| unclear | unclear | low | unclear | high | low | 1 | 0 | 0 |
| unclear | high | unclear | unclear | high | low | 0 | 1 | 0 |
| unclear | low | unclear | unclear | high | low | 1 | 0 | 0 |
| unclear | unclear | unclear | unclear | high | low | 1 | 0 | 0 |
| unclear | high | high | high | low | low | 0 | 0 | 1 |
| unclear | low | high | high | low | low | 0 | 1 | 0 |
| unclear | unclear | high | high | low | low | 0 | 1 | 0 |
| unclear | high | low | high | low | low | 0 | 1 | 0 |
| unclear | low | low | high | low | low | 1 | 0 | 0 |
| unclear | unclear | low | high | low | low | 1 | 0 | 0 |
| unclear | high | unclear | high | low | low | 0 | 1 | 0 |
| unclear | low | unclear | high | low | low | 1 | 0 | 0 |
| unclear | unclear | unclear | high | low | low | 1 | 0 | 0 |
| unclear | high | high | low | low | low | 0 | 1 | 0 |
| unclear | low | high | low | low | low | 1 | 0 | 0 |
| unclear | unclear | high | low | low | low | 1 | 0 | 0 |
| unclear | high | low | low | low | low | 1 | 0 | 0 |
| unclear | low | low | low | low | low | 1 | 0 | 0 |
| unclear | unclear | low | low | low | low | 1 | 0 | 0 |
| unclear | high | unclear | low | low | low | 1 | 0 | 0 |
| unclear | low | unclear | low | low | low | 1 | 0 | 0 |
| unclear | unclear | unclear | low | low | low | 1 | 0 | 0 |
| unclear | high | high | unclear | low | low | 0 | 1 | 0 |
| unclear | low | high | unclear | low | low | 1 | 0 | 0 |
| unclear | unclear | high | unclear | low | low | 1 | 0 | 0 |
| unclear | high | low | unclear | low | low | 1 | 0 | 0 |
| unclear | low | low | unclear | low | low | 1 | 0 | 0 |
| unclear | unclear | low | unclear | low | low | 1 | 0 | 0 |
| unclear | high | unclear | unclear | low | low | 1 | 0 | 0 |
| unclear | low | unclear | unclear | low | low | 1 | 0 | 0 |
| unclear | unclear | unclear | unclear | low | low | 1 | 0 | 0 |
| unclear | high | high | high | unclear | low | 0 | 0 | 1 |
| unclear | low | high | high | unclear | low | 0 | 1 | 0 |
| unclear | unclear | high | high | unclear | low | 0 | 1 | 0 |
| unclear | high | low | high | unclear | low | 0 | 1 | 0 |
| unclear | low | low | high | unclear | low | 1 | 0 | 0 |
| unclear | unclear | low | high | unclear | low | 1 | 0 | 0 |
| unclear | high | unclear | high | unclear | low | 0 | 1 | 0 |
| unclear | low | unclear | high | unclear | low | 1 | 0 | 0 |
| unclear | unclear | unclear | high | unclear | low | 1 | 0 | 0 |
| unclear | high | high | low | unclear | low | 0 | 1 | 0 |
| unclear | low | high | low | unclear | low | 1 | 0 | 0 |
| unclear | unclear | high | low | unclear | low | 1 | 0 | 0 |
| unclear | high | low | low | unclear | low | 1 | 0 | 0 |
| unclear | low | low | low | unclear | low | 1 | 0 | 0 |
| unclear | unclear | low | low | unclear | low | 1 | 0 | 0 |
| unclear | high | unclear | low | unclear | low | 1 | 0 | 0 |
| unclear | low | unclear | low | unclear | low | 1 | 0 | 0 |
| unclear | unclear | unclear | low | unclear | low | 1 | 0 | 0 |
| unclear | high | high | unclear | unclear | low | 0 | 1 | 0 |
| unclear | low | high | unclear | unclear | low | 1 | 0 | 0 |
| unclear | unclear | high | unclear | unclear | low | 1 | 0 | 0 |
| unclear | high | low | unclear | unclear | low | 1 | 0 | 0 |
| unclear | low | low | unclear | unclear | low | 1 | 0 | 0 |
| unclear | unclear | low | unclear | unclear | low | 1 | 0 | 0 |
| unclear | high | unclear | unclear | unclear | low | 1 | 0 | 0 |
| unclear | low | unclear | unclear | unclear | low | 1 | 0 | 0 |
| unclear | unclear | unclear | unclear | unclear | low | 1 | 0 | 0 |

Table S7. Conditional probability table: Risk of bias
