# Supplementary material for: The Use of Bayesian Networks to Assess the Quality of Evidence from Research Synthesis: 1
Source: PLoS One. 2015 Apr 2;10(4):e0114497. doi: 10.1371/journal.pone.0114497 (PMC4383525; doi:10.1371/journal.pone.0114497)
Supplement: S8 Table — (DOCX) [file pone.0114497.s009.docx]

| Overlapping study CIs | substantial | | | | Some overlap | | | | No overlap | | | | unreported | | | |
| --- | --- | --- | --- | --- | --- | --- | --- | --- | --- | --- | --- | --- | --- | --- | --- | --- |
| I2 (Tau) | high | moderate | low | unreported | high | moderate | low | unreported | high | moderate | low | unreported | high | moderate | low | unreported |
| consistent | 0.7 | 0.8 | 1 | 1 | 0.35 | 0.5 | 0.65 | 0.5 | 0 | 0.2 | 0.3 | 0 | 0 | 0.5 | 1 | 1 |
| inconsistent | 0.3 | 0.2 | 0 | 0 | 0.65 | 0.5 | 0.35 | 0.5 | 1 | 0.8 | 0.7 | 1 | 1 | 0.5 | 0 | 0 |

Table S8. Conditional probability table: Consistency
