# Supplementary material for: The Use of Bayesian Networks to Assess the Quality of Evidence from Research Synthesis: 1
Source: PLoS One. 2015 Apr 2;10(4):e0114497. doi: 10.1371/journal.pone.0114497 (PMC4383525; doi:10.1371/journal.pone.0114497)
Supplement: S9 Table — (DOCX) [file pone.0114497.s010.docx]

.

| Statistical test of heterogeneity | significant | not significant | unclear |
| --- | --- | --- | --- |
| high | 1 | 0 | 0.5 |
| low | 0 | 1 | 0.5 |

Table S9. Conditional probability table: Strength of evidence
