# Supplementary material for: The Use of Bayesian Networks to Assess the Quality of Evidence from Research Synthesis: 1
Source: PLoS One. 2015 Apr 2;10(4):e0114497. doi: 10.1371/journal.pone.0114497 (PMC4383525; doi:10.1371/journal.pone.0114497)
Supplement: S10 Table — (DOCX) [file pone.0114497.s011.docx]

| Point estimates are consistent | **no** | | **yes** | |
| --- | --- | --- | --- | --- |
| Consistency of effect direction | **yes** | **no** | **yes** | **no** |
| Wide dispersion | 0.5 | 1 | 0 | 0 |
| Some dispersion | 0.5 | 0 | 0 | 0 |
| No dispersion | 0 | 0 | 1 | 1 |

Table S10. Conditional probability table: Study distribution
