# Supplementary material for: The Use of Bayesian Networks to Assess the Quality of Evidence from Research Synthesis: 1
Source: PLoS One. 2015 Apr 2;10(4):e0114497. doi: 10.1371/journal.pone.0114497 (PMC4383525; doi:10.1371/journal.pone.0114497)
Supplement: S11 Table — (DOCX) [file pone.0114497.s012.docx]

| Study distribution | wide dispersion | | | | Some dispersion | | | | No dispersion | | | |
| --- | --- | --- | --- | --- | --- | --- | --- | --- | --- | --- | --- | --- |
| Consistency | consistent | | inconsistent | | consistent | | inconsistent | | consistent | | inconsistent | |
| Strength of evidence | high | low | high | low | high | low | high | low | high | low | high | low |
| no | 0 | 0 | 0 | 0 | 0.2 | 0.2 | 0 | 0 | 1 | 0.95 | 0.8 | 0.8 |
| serious | 0.2 | 0.2 | 0 | 0.05 | 0.8 | 0.8 | 1 | 1 | 0 | 0.05 | 0.2 | 0.2 |
| Very serious | 0.8 | 0.8 | 1 | 0.95 | 0 | 0 | 0 | 0 | 0 | 0 | 0 | 0 |

Table S11. Conditional probability table: Inconsistency
