# Supplementary material for: The Use of Bayesian Networks to Assess the Quality of Evidence from Research Synthesis: 1
Source: PLoS One. 2015 Apr 2;10(4):e0114497. doi: 10.1371/journal.pone.0114497 (PMC4383525; doi:10.1371/journal.pone.0114497)
Supplement: S12 Table — (DOCX) [file pone.0114497.s013.docx]

| Applicability of population | high | | | | | | applicable | | | | | | poor | | | | | |
| --- | --- | --- | --- | --- | --- | --- | --- | --- | --- | --- | --- | --- | --- | --- | --- | --- | --- | --- |
| Applicability of intervention | high | | applicable | | poor | | high | | applicable | | poor | | high | | applicable | | poor | |
| Outcome timeframe | insufficient | sufficient | insufficient | sufficient | insufficient | sufficient | insufficient | sufficient | insufficient | sufficient | insufficient | sufficient | insufficient | sufficient | insufficient | sufficient | insufficient | sufficient |
| high | 0.8 | 1 | 0.6 | 0.8 | 0.3 | 0.5 | 0.6 | 0.8 | 0.5 | 0.7 | 0.2 | 0.4 | 0.2 | 0.4 | 0.1 | 0.3 | 0 | 0 |
| low | 0.2 | 0 | 0.4 | 0.2 | 0.7 | 0.5 | 0.4 | 0.2 | 0.5 | 0.3 | 0.8 | 0.6 | 0.8 | 0.6 | 0.9 | 0.7 | 1 | 1 |

Table S12. Conditional probability table: Applicability
