# Supplementary material for: The Use of Bayesian Networks to Assess the Quality of Evidence from Research Synthesis: 1
Source: PLoS One. 2015 Apr 2;10(4):e0114497. doi: 10.1371/journal.pone.0114497 (PMC4383525; doi:10.1371/journal.pone.0114497)
Supplement: S13 Table — (DOCX) [file pone.0114497.s014.docx]

| Not a surrogate outcome | no | | | | yes | | | |
| --- | --- | --- | --- | --- | --- | --- | --- | --- |
| Applicability | high | | low | | high | | low | |
| Direct comparison | no | yes | no | yes | no | yes | no | yes |
| no | 0 | 0 | 0 | 0 | 0.9 | 1 | 0 | 0 |
| serious | 0.6 | 0.7 | 0 | 0.1 | 0.1 | 0 | 0.8 | 0.9 |
| Very serious | 0.4 | 0.3 | 1 | 0.9 | 0 | 0 | 0.2 | 0.1 |

Table S13. Conditional probability table: Indirectness
