# Supplementary material for: The Use of Bayesian Networks to Assess the Quality of Evidence from Research Synthesis: 1
Source: PLoS One. 2015 Apr 2;10(4):e0114497. doi: 10.1371/journal.pone.0114497 (PMC4383525; doi:10.1371/journal.pone.0114497)
Supplement: S14 Table — (DOCX) [file pone.0114497.s015.docx]

| Study size | large | | | | | | | | | moderate | | | | | | | | | small | | | | | | | | |
| --- | --- | --- | --- | --- | --- | --- | --- | --- | --- | --- | --- | --- | --- | --- | --- | --- | --- | --- | --- | --- | --- | --- | --- | --- | --- | --- | --- |
| No of studies | high | | | moderate | | | low | | | high | | | moderate | | | low | | | high | | | moderate | | | low | | |
| Common events | no | yes | na | no | yes | na | no | yes | na | no | yes | na | no | yes | na | no | yes | na | no | yes | na | no | yes | na | no | yes | na |
| high | 0.3 | 1 | 1 | 0 | 0.85 | 0.85 | 0 | 0.8 | 0.8 | 0 | 0.57 | 0.57 | 0 | 0.425 | 0.425 | 0 | 0.4 | 0.4 | 0 | 0.15 | 0.15 | 0 | 0 | 0 | 0 | 0 | 0 |
| intermediate | 0.7 | 0 | 0 | 0.4 | 0.15 | 0.15 | 0.3 | 0.2 | 0.2 | 0.3 | 0.35 | 0.35 | 0.2 | 0.375 | 0.375 | 0.2 | 0.15 | 0.15 | 0.1 | 0.7 | 0.7 | 0.1 | 0.6 | 0.6 | 0 | 0.05 | 0.05 |
| low | 0 | 0 | 0 | 0.6 | 0 | 0 | 0.7 | 0 | 0 | 0.7 | 0.07 | 0.07 | 0.8 | 0.2 | 0.2 | 0.8 | 0.45 | 0.45 | 0.9 | 0.15 | 0.15 | 0.9 | 0.4 | 0.4 | 1 | 0.95 | 0.95 |

Table S14. Conditional probability table: Statistical information
