# Supplementary material for: The Use of Bayesian Networks to Assess the Quality of Evidence from Research Synthesis: 1
Source: PLoS One. 2015 Apr 2;10(4):e0114497. doi: 10.1371/journal.pone.0114497 (PMC4383525; doi:10.1371/journal.pone.0114497)
Supplement: S15 Table — (DOCX) [file pone.0114497.s016.docx]

| Statistical information | high | | | | | | | | intermediate | | | | | | | | low | | | | | | | |
| --- | --- | --- | --- | --- | --- | --- | --- | --- | --- | --- | --- | --- | --- | --- | --- | --- | --- | --- | --- | --- | --- | --- | --- | --- |
| Benefit only | no | | | | yes | | | | no | | | | yes | | | | no | | | | yes | | | |
| Guideline or SR | G | | SR | | G | | SR | | G | | SR | | G | | SR | | G | | SR | | G | | SR | |
| No serious harm | no | yes | no | yes | no | yes | no | yes | no | yes | no | yes | no | yes | no | yes | no | yes | no | yes | no | yes | no | yes |
| no | 0 | 0.3 | 0 | 0 | 1 | 1 | 1 | 1 | 0 | 0.35 | 0.35 | 0.35 | 0.9 | 1 | 1 | 1 | 0 | 0 | 0 | 0 | 0 | 0.2 | 0.2 | 0.2 |
| serious | 1 | 0.7 | 1 | 1 | 0 | 0 | 0 | 0 | 0.7 | 0.65 | 0.65 | 0.65 | 0.1 | 0 | 0 | 0 | 0.1 | 0.6 | 0.6 | 0.6 | 1 | 0.8 | 0.8 | 0.8 |
| Very serious | 0 | 0 | 0 | 0 | 0 | 0 | 0 | 0 | 0.3 | 0 | 0 | 0 | 0 | 0 | 0 | 0 | 0.9 | 0.4 | 0.4 | 0.4 | 0 | 0 | 0 | 0 |

Table S15. Conditional probability table: Imprecision
