# Supplementary material for: The Use of Bayesian Networks to Assess the Quality of Evidence from Research Synthesis: 1
Source: PLoS One. 2015 Apr 2;10(4):e0114497. doi: 10.1371/journal.pone.0114497 (PMC4383525; doi:10.1371/journal.pone.0114497)
Supplement: S16 Table — (DOCX) [file pone.0114497.s017.docx]

| Discrepancies between studies | Statistical information | No industry influence | Search integrity | strongly suspected | undetected |
| --- | --- | --- | --- | --- | --- |
| yes | high | no | high | 1 | 0 |
| yes | high | no | low | 1 | 0 |
| yes | high | yes | high | 0.8 | 0.2 |
| yes | high | yes | low | 1 | 0 |
| yes | intermediate | no | high | 1 | 0 |
| yes | intermediate | no | low | 1 | 0 |
| yes | intermediate | yes | high | 0.8 | 0.2 |
| yes | intermediate | yes | low | 1 | 0 |
| yes | low | no | high | 1 | 0 |
| yes | low | no | low | 1 | 0 |
| yes | low | yes | high | 0.8 | 0.2 |
| yes | low | yes | low | 1 | 0 |
| no | high | no | high | 0.3 | 0.7 |
| no | high | no | low | 0.5 | 0.5 |
| no | high | yes | high | 0 | 1 |
| no | high | yes | low | 0.1 | 0.9 |
| no | intermediate | no | high | 0.4 | 0.6 |
| no | intermediate | no | low | 0.6 | 0.4 |
| no | intermediate | yes | high | 0.1 | 0.9 |
| no | intermediate | yes | low | 0.2 | 0.8 |
| no | low | no | high | 0.6 | 0.4 |
| no | low | no | low | 0.7 | 0.3 |
| no | low | yes | high | 0.3 | 0.7 |
| no | low | yes | low | 0.5 | 0.5 |

Table S16. Conditional probability table: Publication bias
