# Supplementary material for: The Use of Bayesian Networks to Assess the Quality of Evidence from Research Synthesis: 1
Source: PLoS One. 2015 Apr 2;10(4):e0114497. doi: 10.1371/journal.pone.0114497 (PMC4383525; doi:10.1371/journal.pone.0114497)
Supplement: S17 Table — (DOCX) [file pone.0114497.s018.docx]

| **Scenario** | **Risk of bias** | **Inconsistency** | **Indirectness** | **Imprecision** | **Publication bias** | **Overall reliability** |
| --- | --- | --- | --- | --- | --- | --- |
| **One** | **No 100%**  **Serious 0%**  **V serious 0%** | **No 89.5%**  **Serious 10.5%**  **V serious 0%** | **No 100%**  **Serious 0%**  **V serious 0%** | **No 100%**  **Serious 0%**  **V serious 0%** | **Not detected 100%**  **Strongly suspected 0%** | **High 89.5%**  **Moderate 10.5%**  **Low 0%**  **V low 0%** |
| **Two** | **No 100%**  **Serious 0%**  **V serious 0%** | **No 89.5%**  **Serious 10.5%**  **V serious 0%** | **No 100%**  **Serious 0%**  **V serious 0%** | **No 28%**  **Serious 72%**  **V serious 0%** | **Not detected 72%**  **Strongly suspected 28%** | **High 19.3%**  **Moderate 53.1%**  **Low 25.3%**  **V low 2.3%** |
| **Three** | **No 50%**  **Serious 50%**  **V serious 0%** | **No 89.5%**  **Serious 10.5%**  **V serious 0%** | **No 30%**  **Serious 49%**  **V serious 21%** | **No 100%**  **Serious 0%**  **V serious 0%** | **Not detected 100%**  **Strongly suspected 0%** | **High 13.4%**  **Moderate 36.9%**  **Low 35.5%**  **V low 14.2%** |
| **Four** | **No 100%**  **Serious 0%**  **V serious 0%** | **No 63%**  **Serious 34%**  **V serious 3%** | **No 100%**  **Serious 0%**  **V serious 0%** | **No 84%**  **Serious 16%**  **V serious 0%** | **Not detected 78.3%**  **Strongly suspected 21.7%** | **High 44.3%**  **Moderate 37.6%**  **Low 14.5%**  **V low 3.6%** |
| **Five** | **No 100%**  **Serious 0%**  **V serious 0%** | **No 7%**  **Serious 59%**  **V serious 34%** | **No 30%**  **Serious 49%**  **V serious 21%** | **No 68%**  **Serious 32%**  **V serious 0%** | **Not detected 52%**  **Strongly suspected 48%** | **High 0.8%**  **Moderate 9%**  **Low 25.1%**  **V low 64.9%** |

Table S17. Probabilities of bias and overall reliability for five scenerios.
